# (Methanol-κ*O*)(2-methyl-3-nitro­benzoato-κ*O*)triphenyl­tin(IV)

**DOI:** 10.1107/S1600536810033623

**Published:** 2010-08-28

**Authors:** Foo Win Yip, Siang Guan Teoh, Bohari M. Yamin, Seik Weng Ng

**Affiliations:** a Department of Chemical Science, Faculty of Science, Universiti Tunku Abdul Rahman, 31900 Kampar, Malaysia; b School of Chemical Sciences, Universiti Sains Malaysia, 11800 Penang, Malaysia; c School of Chemical Sciences and Food Technology, Faculty of Science and Technology, Universiti Kebangbaan Malaysia, 43600 Bangi, Malaysia; d Department of Chemistry, University of Malaya, 50603 Kuala Lumpur, Malaysia

## Abstract

The five-coordinate Sn atom in the title compound, [Sn(C_6_H_5_)_3_(C_8_H_6_NO_4_)(CH_3_OH)], exists in a *trans*-C_3_SnO_2_ trigonal-bipyramidal coordination polyhedron of which the O atoms of the methanol mol­ecule and carboxyl­ate group occupy the apical sites. In the crystal, adjacent mol­ecules are linked by inter­molecular O—H⋯O inter­actions, generating a helical hydrogen-bonded chain running along the *b* axis.

## Related literature

For other methanol/ethanol-coordinated triphenyl­tin carboxyl­ates, see: Alcock & Roe (1989 ▶); Gao *et al.* (2006 ▶); Lo & Ng (2009 ▶); Ma *et al.* (2004 ▶); Ng (1998 ▶, 1999 ▶); Wang *et al.* (2007 ▶); Yeap & Teoh (2003 ▶); Yin *et al.* (2002 ▶).
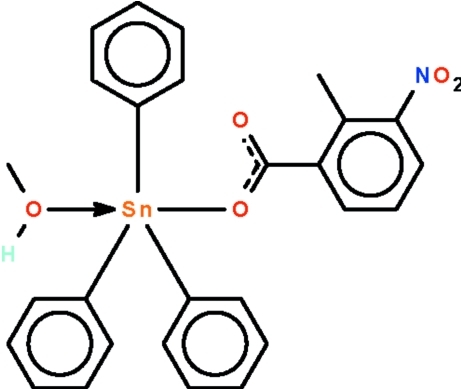


## Experimental

### 

#### Crystal data

[Sn(C_6_H_5_)_3_(C_8_H_6_NO_4_)(CH_4_O)]
*M* *_r_* = 562.17
Monoclinic, 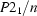

*a* = 10.8385 (13) Å
*b* = 14.8791 (18) Å
*c* = 15.8243 (19) Å
β = 94.004 (2)°
*V* = 2545.7 (5) Å^3^
*Z* = 4
Mo *K*α radiation
μ = 1.04 mm^−1^
*T* = 293 K
0.40 × 0.25 × 0.10 mm

#### Data collection

Bruker SMART diffractometer
Absorption correction: multi-scan (*SADABS*; Sheldrick, 1996 ▶) *T*_min_ = 0.681, *T*_max_ = 0.903
15231 measured reflections
5739 independent reflections
4515 reflections with *I* > 2σ(*I*)
*R*_int_ = 0.024

#### Refinement

*R*[*F* ^2^ > 2σ(*F* ^2^)] = 0.041
*wR*(*F* ^2^) = 0.115
*S* = 1.08
5739 reflections
313 parameters
1 restraint
H atoms treated by a mixture of independent and constrained refinement
Δρ_max_ = 1.15 e Å^−3^
Δρ_min_ = −0.29 e Å^−3^

### 

Data collection: *SMART* (Bruker, 2002 ▶); cell refinement: *SAINT* (Bruker, 2002 ▶); data reduction: *SAINT*; program(s) used to solve structure: *SHELXS97* (Sheldrick, 2008 ▶); program(s) used to refine structure: *SHELXL97* (Sheldrick, 2008 ▶); molecular graphics: *X-SEED* (Barbour, 2001 ▶); software used to prepare material for publication: *publCIF* (Westrip, 2010 ▶).

## Supplementary Material

Crystal structure: contains datablocks global, I. DOI: 10.1107/S1600536810033623/bt5330sup1.cif

Structure factors: contains datablocks I. DOI: 10.1107/S1600536810033623/bt5330Isup2.hkl

Additional supplementary materials:  crystallographic information; 3D view; checkCIF report

## Figures and Tables

**Table 1:** Selected bond lengths (Å)

Sn1—C1	2.118 (4)
Sn1—C7	2.110 (4)
Sn1—C13	2.121 (4)
Sn1—O1	2.146 (2)
Sn1—O5	2.410 (3)

**Table 2:** Hydrogen-bond geometry (Å, °)

*D*—H⋯*A*	*D*—H	H⋯*A*	*D*⋯*A*	*D*—H⋯*A*
O5—H5⋯O2^i^	0.85 (1)	1.87 (2)	2.676 (4)	158 (5)

Symmetry code: (i) 
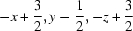
.

**Fig. 1.**
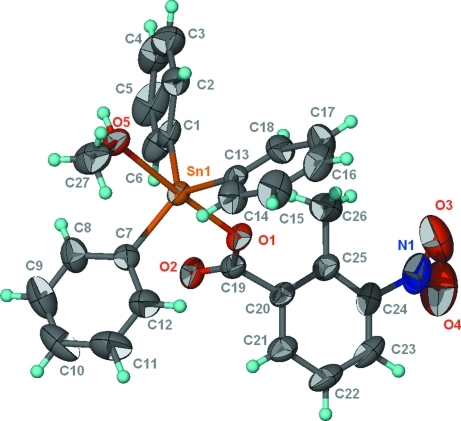
Anisotropic displacement ellipsoid plot (Barbour, 2001) of the title compound at the 50% probability level; hydrogen atoms are drawn as spheres of arbitrary radius.

**Fig. 2.**
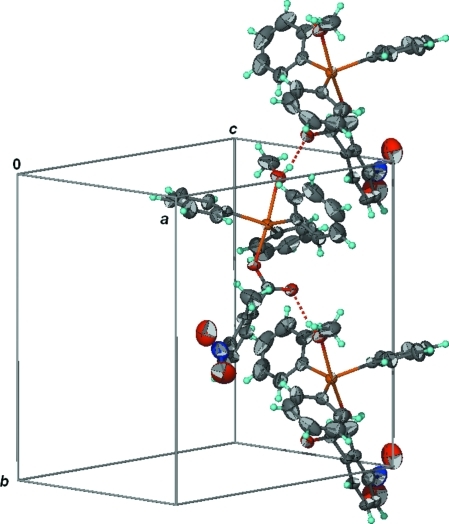
Hydrogen-bonded chain structure.

## supplementary crystallographic information

### Comment

Triphenyltin(IV) carboxylates generally exist as tetrahedral molecules in which the carboxylate anion is only unidentate to the tin atom. However, there are examples of such compounds having an alcohol molecule into its coordination sphere, as noted in several examples (Alcock & Roe, 1989, Gao *et al.*, 2006; Lo & Ng, 2009; Ma *et al.*, 2004; Ng, 1998; Ng, 1999; Wang *et al.*, 2007; Yeap & Teoh, 2003; Yin *et al.* 2002). The alcohol (either methanol or ethanol) serves as the reaction medium. The condensation of triphenyltin hydroxide and 2-methyl-3-nitrobenzoic acid in methanol gave the expected product as a methanol-coordinated compound (Scheme I, Fig. 1). The five-coordinate tin atom in Sn(C_6_H_5_)_3_(CH_4_O)(C_8_H_6_NO_4_) exists in a *trans*-C_3_SnO_2_ trigonal bipyramidal coordination polyhedron for which the O atoms of the methanol and carboxylate occupy the apical sites. As found in the other examples, the tin-oxygen_alcohol_ bond is longer than the tin-oxygen_carboxylate_ bond (Table 1). However, the C_3_Sn girdle is nearly planar [Σ_angles_ 358.6 (5) °]. Adjacent molecules are linked by an O–H···O interaction to generate a helical hydrogen-bonded chain running along the *b*-axis of the monoclinic unit cell (Fig. 2, Table 2).

### Experimental

Triphenyltin hydroxide (0.73 g, 2 mmol) and 2-methyl-3-nitrobenzoic acid (0.36 g, 2 mmole) were heated in methanol (50 ml) for an hour. The mixture was filtered to give a clear yellow solution. The filtrate was set aside for the formation of crystals, which were isolated in 80% yield; m.p. 394.5 K. CHN&Sn elemental analysis for C_27_H_25_N_1_O_5_Sn: C 57.53, H 4.04, N 2.45, Sn 21.02%. Calc.: C 57.68, H 4.48, N 2.49, Sn 21.11%.

### Refinement

Carbon-bound H-atoms were placed in calculated positions (C—H 0.93 to 0.97 Å) and were included in the refinement in the riding model approximation, with *U*(H) set to 1.2 to 1.5*U*(C).

The methanol H-atom was located in a difference Fourier map, and was refined isotropically  with a distance restraint of O–H 0.85±0.01 Å.

The final difference Fourier map had two peaks in the vicinity of Sn1.

### Crystal data

**Table:**

[Sn(C_6_H_5_)_3_(C_8_H_6_NO_4_)(CH_4_O)]	*F*(000) = 1136
*M**_r_* = 562.17	*D*_x_ = 1.467 Mg m^−^^3^
Monoclinic, *P*2_1_/*n*	Mo *K*α radiation, λ = 0.71073 Å
Hall symbol: -P 2yn	Cell parameters from 918 reflections
*a* = 10.8385 (13) Å	θ = 2.7–25.1°
*b* = 14.8791 (18) Å	µ = 1.04 mm^−^^1^
*c* = 15.8243 (19) Å	*T* = 293 K
β = 94.004 (2)°	Block, yellow
*V* = 2545.7 (5) Å^3^	0.40 × 0.25 × 0.10 mm
*Z* = 4	

### Data collection

**Table:**

Bruker SMART diffractometer	5739 independent reflections
Radiation source: fine-focus sealed tube	4515 reflections with *I* > 2σ(*I*)
graphite	*R*_int_ = 0.024
ω scans	θ_max_ = 27.5°, θ_min_ = 1.9°
Absorption correction: multi-scan (*SADABS*; Sheldrick, 1996)	*h* = −14→13
*T*_min_ = 0.681, *T*_max_ = 0.903	*k* = −18→19
15231 measured reflections	*l* = −20→10

### Refinement

**Table:**

Refinement on *F*^2^	Primary atom site location: structure-invariant direct methods
Least-squares matrix: full	Secondary atom site location: difference Fourier map
*R*[*F*^2^ > 2σ(*F*^2^)] = 0.041	Hydrogen site location: inferred from neighbouring sites
*wR*(*F*^2^) = 0.115	H atoms treated by a mixture of independent and constrained refinement
*S* = 1.08	*w* = 1/[σ^2^(*F*_o_^2^) + (0.0581*P*)^2^ + 1.4418*P*] where *P* = (*F*_o_^2^ + 2*F*_c_^2^)/3
5739 reflections	(Δ/σ)_max_ = 0.001
313 parameters	Δρ_max_ = 1.15 e Å^−^^3^
1 restraint	Δρ_min_ = −0.29 e Å^−^^3^

### Fractional atomic coordinates and isotropic or equivalent isotropic displacement parameters (Å^2^)

**Table:**

	*x*	*y*	*z*	*U*_iso_*/*U*_eq_	
Sn1	0.63268 (2)	0.197738 (16)	0.687848 (16)	0.04465 (10)	
O1	0.6379 (3)	0.32447 (16)	0.62345 (18)	0.0566 (7)	
O2	0.7358 (3)	0.40787 (19)	0.72344 (18)	0.0635 (7)	
O3	0.7290 (6)	0.5035 (5)	0.3367 (3)	0.140 (2)	
O4	0.7949 (6)	0.6315 (4)	0.3673 (4)	0.160 (2)	
O5	0.6168 (3)	0.05094 (19)	0.7505 (2)	0.0617 (7)	
H5	0.679 (3)	0.016 (3)	0.758 (3)	0.089 (17)*	
N1	0.7459 (5)	0.5650 (4)	0.3876 (4)	0.0993 (15)	
C1	0.8227 (4)	0.1658 (3)	0.6847 (3)	0.0530 (9)	
C2	0.8560 (5)	0.0862 (3)	0.6479 (3)	0.0705 (12)	
H2	0.7951	0.0485	0.6236	0.085*	
C3	0.9784 (7)	0.0619 (5)	0.6469 (4)	0.103 (2)	
H3	0.9998	0.0074	0.6231	0.124*	
C4	1.0676 (6)	0.1176 (7)	0.6805 (5)	0.118 (3)	
H4	1.1502	0.1008	0.6801	0.141*	
C5	1.0378 (5)	0.1978 (5)	0.7148 (5)	0.108 (2)	
H5A	1.1001	0.2360	0.7368	0.130*	
C6	0.9150 (4)	0.2230 (3)	0.7172 (3)	0.0756 (14)	
H6	0.8946	0.2780	0.7405	0.091*	
C7	0.5464 (4)	0.2443 (3)	0.7950 (2)	0.0512 (9)	
C8	0.5951 (5)	0.2284 (4)	0.8764 (3)	0.0766 (13)	
H8	0.6674	0.1951	0.8851	0.092*	
C9	0.5383 (7)	0.2609 (5)	0.9442 (4)	0.0999 (19)	
H9	0.5728	0.2496	0.9986	0.120*	
C10	0.4323 (7)	0.3094 (4)	0.9338 (4)	0.101 (2)	
H10	0.3944	0.3312	0.9806	0.121*	
C11	0.3822 (6)	0.3257 (4)	0.8546 (5)	0.1000 (19)	
H11	0.3093	0.3585	0.8471	0.120*	
C12	0.4384 (5)	0.2940 (3)	0.7847 (4)	0.0764 (14)	
H12	0.4035	0.3061	0.7306	0.092*	
C13	0.5186 (3)	0.1510 (2)	0.5826 (2)	0.0473 (8)	
C14	0.3968 (4)	0.1301 (3)	0.5878 (3)	0.0704 (12)	
H14	0.3631	0.1296	0.6402	0.085*	
C15	0.3226 (5)	0.1094 (4)	0.5146 (4)	0.0900 (17)	
H15	0.2396	0.0954	0.5187	0.108*	
C16	0.3702 (6)	0.1096 (3)	0.4381 (4)	0.0908 (18)	
H16	0.3198	0.0972	0.3895	0.109*	
C17	0.4918 (7)	0.1280 (4)	0.4322 (3)	0.0914 (17)	
H17	0.5257	0.1258	0.3799	0.110*	
C18	0.5658 (5)	0.1501 (3)	0.5044 (3)	0.0701 (12)	
H18	0.6486	0.1644	0.4997	0.084*	
C19	0.6831 (4)	0.3978 (3)	0.6536 (3)	0.0520 (9)	
C20	0.6661 (4)	0.4765 (2)	0.5946 (2)	0.0493 (9)	
C21	0.5921 (4)	0.5464 (3)	0.6191 (3)	0.0634 (11)	
H21	0.5564	0.5429	0.6708	0.076*	
C22	0.5704 (5)	0.6205 (3)	0.5691 (4)	0.0773 (15)	
H22	0.5198	0.6666	0.5861	0.093*	
C23	0.6240 (5)	0.6255 (3)	0.4942 (4)	0.0747 (14)	
H23	0.6111	0.6757	0.4597	0.090*	
C24	0.6958 (4)	0.5577 (3)	0.4699 (3)	0.0660 (11)	
C25	0.7200 (4)	0.4793 (3)	0.5179 (3)	0.0589 (10)	
C26	0.8001 (6)	0.4023 (4)	0.4908 (4)	0.0979 (18)	
H26	0.8447	0.3771	0.5397	0.147*	
H26B	0.8576	0.4242	0.4522	0.147*	
H26C	0.7486	0.3569	0.4634	0.147*	
C27	0.5104 (5)	0.0158 (4)	0.7849 (4)	0.0936 (18)	
H27A	0.5287	0.0021	0.8437	0.140*	
H27B	0.4849	−0.0380	0.7550	0.140*	
H27C	0.4450	0.0594	0.7794	0.140*	

### Atomic displacement parameters (Å^2^)

**Table:**

	*U*^11^	*U*^22^	*U*^33^	*U*^12^	*U*^13^	*U*^23^
Sn1	0.04851 (16)	0.03856 (15)	0.04577 (16)	0.00015 (10)	−0.00464 (10)	0.00265 (10)
O1	0.0819 (19)	0.0289 (12)	0.0569 (16)	−0.0078 (12)	−0.0114 (14)	0.0025 (11)
O2	0.087 (2)	0.0475 (15)	0.0527 (16)	−0.0111 (14)	−0.0160 (15)	0.0024 (13)
O3	0.152 (5)	0.195 (6)	0.075 (3)	−0.012 (4)	0.021 (3)	−0.002 (4)
O4	0.187 (5)	0.158 (5)	0.139 (5)	−0.054 (4)	0.028 (4)	0.063 (4)
O5	0.0636 (18)	0.0493 (15)	0.073 (2)	0.0051 (13)	0.0088 (15)	0.0157 (14)
N1	0.099 (3)	0.111 (4)	0.087 (4)	−0.007 (3)	0.003 (3)	0.037 (3)
C1	0.050 (2)	0.054 (2)	0.055 (2)	0.0003 (17)	0.0007 (17)	0.0189 (19)
C2	0.078 (3)	0.069 (3)	0.066 (3)	0.012 (2)	0.018 (2)	0.012 (2)
C3	0.098 (5)	0.114 (5)	0.103 (5)	0.041 (4)	0.040 (4)	0.027 (4)
C4	0.061 (4)	0.165 (8)	0.130 (6)	0.031 (4)	0.031 (4)	0.067 (6)
C5	0.054 (3)	0.130 (6)	0.140 (6)	−0.019 (3)	−0.005 (3)	0.047 (5)
C6	0.063 (3)	0.069 (3)	0.093 (4)	−0.010 (2)	−0.011 (3)	0.024 (3)
C7	0.056 (2)	0.045 (2)	0.052 (2)	−0.0054 (16)	0.0022 (17)	−0.0023 (17)
C8	0.075 (3)	0.100 (4)	0.053 (3)	−0.004 (3)	−0.007 (2)	−0.002 (3)
C9	0.108 (5)	0.135 (6)	0.056 (3)	−0.030 (4)	0.007 (3)	−0.015 (3)
C10	0.115 (5)	0.104 (5)	0.090 (5)	−0.041 (4)	0.048 (4)	−0.040 (4)
C11	0.085 (4)	0.091 (4)	0.128 (6)	0.008 (3)	0.033 (4)	−0.020 (4)
C12	0.078 (3)	0.077 (3)	0.074 (3)	0.015 (2)	0.006 (3)	−0.005 (2)
C13	0.058 (2)	0.0339 (17)	0.049 (2)	0.0034 (15)	−0.0081 (17)	−0.0018 (15)
C14	0.067 (3)	0.077 (3)	0.066 (3)	−0.015 (2)	−0.010 (2)	−0.003 (2)
C15	0.077 (3)	0.094 (4)	0.095 (4)	−0.026 (3)	−0.024 (3)	−0.001 (3)
C16	0.127 (5)	0.061 (3)	0.077 (4)	−0.014 (3)	−0.044 (4)	−0.006 (3)
C17	0.124 (5)	0.096 (4)	0.052 (3)	0.005 (4)	−0.011 (3)	−0.015 (3)
C18	0.076 (3)	0.076 (3)	0.058 (3)	0.008 (2)	−0.003 (2)	−0.013 (2)
C19	0.059 (2)	0.045 (2)	0.052 (2)	0.0048 (17)	−0.0002 (19)	−0.0010 (17)
C20	0.061 (2)	0.0312 (16)	0.054 (2)	−0.0017 (15)	−0.0105 (18)	−0.0013 (15)
C21	0.072 (3)	0.042 (2)	0.074 (3)	0.0043 (18)	−0.010 (2)	−0.0046 (19)
C22	0.085 (3)	0.042 (2)	0.101 (4)	0.015 (2)	−0.020 (3)	0.002 (2)
C23	0.078 (3)	0.044 (2)	0.097 (4)	0.001 (2)	−0.031 (3)	0.014 (2)
C24	0.071 (3)	0.067 (3)	0.059 (3)	−0.008 (2)	−0.007 (2)	0.015 (2)
C25	0.069 (3)	0.044 (2)	0.062 (3)	0.0004 (18)	0.001 (2)	0.0051 (18)
C26	0.122 (5)	0.088 (4)	0.088 (4)	0.030 (3)	0.039 (4)	0.010 (3)
C27	0.095 (4)	0.065 (3)	0.124 (5)	0.001 (3)	0.034 (4)	0.022 (3)

### Geometric parameters (Å, °)

**Table:**

Sn1—C1	2.118 (4)	C11—C12	1.383 (8)
Sn1—C7	2.110 (4)	C11—H11	0.9300
Sn1—C13	2.121 (4)	C12—H12	0.9300
Sn1—O1	2.146 (2)	C13—C18	1.371 (6)
Sn1—O5	2.410 (3)	C13—C14	1.364 (6)
O1—C19	1.275 (5)	C14—C15	1.397 (7)
O2—C19	1.218 (4)	C14—H14	0.9300
O3—N1	1.225 (7)	C15—C16	1.349 (8)
O4—N1	1.179 (6)	C15—H15	0.9300
O5—C27	1.410 (6)	C16—C17	1.356 (9)
O5—H5	0.850 (10)	C16—H16	0.9300
N1—C24	1.450 (7)	C17—C18	1.389 (7)
C1—C6	1.384 (6)	C17—H17	0.9300
C1—C2	1.378 (6)	C18—H18	0.9300
C2—C3	1.376 (8)	C19—C20	1.500 (5)
C2—H2	0.9300	C20—C21	1.385 (5)
C3—C4	1.354 (10)	C20—C25	1.384 (6)
C3—H3	0.9300	C21—C22	1.367 (6)
C4—C5	1.360 (10)	C21—H21	0.9300
C4—H4	0.9300	C22—C23	1.358 (8)
C5—C6	1.386 (8)	C22—H22	0.9300
C5—H5A	0.9300	C23—C24	1.346 (7)
C6—H6	0.9300	C23—H23	0.9300
C7—C8	1.378 (6)	C24—C25	1.407 (6)
C7—C12	1.383 (6)	C25—C26	1.517 (6)
C8—C9	1.363 (8)	C26—H26	0.9600
C8—H8	0.9300	C26—H26B	0.9600
C9—C10	1.357 (9)	C26—H26C	0.9600
C9—H9	0.9300	C27—H27A	0.9600
C10—C11	1.351 (10)	C27—H27B	0.9600
C10—H10	0.9300	C27—H27C	0.9600
			
C7—Sn1—C1	125.41 (16)	C11—C12—H12	119.8
C7—Sn1—C13	118.16 (15)	C18—C13—C14	118.4 (4)
C1—Sn1—C13	114.98 (16)	C18—C13—Sn1	118.6 (3)
C7—Sn1—O1	96.93 (13)	C14—C13—Sn1	122.7 (3)
C1—Sn1—O1	97.32 (13)	C13—C14—C15	120.2 (5)
C13—Sn1—O1	87.06 (12)	C13—C14—H14	119.9
C7—Sn1—O5	85.35 (13)	C15—C14—H14	119.9
C1—Sn1—O5	84.49 (13)	C16—C15—C14	120.6 (5)
C13—Sn1—O5	88.38 (12)	C16—C15—H15	119.7
O1—Sn1—O5	175.43 (10)	C14—C15—H15	119.7
C19—O1—Sn1	126.7 (3)	C17—C16—C15	119.7 (5)
C27—O5—Sn1	125.3 (3)	C17—C16—H16	120.1
C27—O5—H5	112 (4)	C15—C16—H16	120.1
Sn1—O5—H5	122 (4)	C16—C17—C18	120.0 (6)
O4—N1—O3	119.9 (7)	C16—C17—H17	120.0
O4—N1—C24	120.7 (7)	C18—C17—H17	120.0
O3—N1—C24	119.2 (5)	C13—C18—C17	120.9 (5)
C6—C1—C2	118.8 (4)	C13—C18—H18	119.5
C6—C1—Sn1	122.1 (4)	C17—C18—H18	119.5
C2—C1—Sn1	119.1 (3)	O2—C19—O1	126.0 (4)
C1—C2—C3	120.8 (6)	O2—C19—C20	119.9 (3)
C1—C2—H2	119.6	O1—C19—C20	114.1 (3)
C3—C2—H2	119.6	C21—C20—C25	120.7 (4)
C4—C3—C2	119.8 (6)	C21—C20—C19	117.4 (4)
C4—C3—H3	120.1	C25—C20—C19	121.8 (3)
C2—C3—H3	120.1	C22—C21—C20	121.5 (5)
C5—C4—C3	120.7 (6)	C22—C21—H21	119.2
C5—C4—H4	119.6	C20—C21—H21	119.2
C3—C4—H4	119.6	C21—C22—C23	118.8 (5)
C4—C5—C6	120.3 (6)	C21—C22—H22	120.6
C4—C5—H5A	119.9	C23—C22—H22	120.6
C6—C5—H5A	119.9	C24—C23—C22	120.0 (4)
C1—C6—C5	119.6 (6)	C24—C23—H23	120.0
C1—C6—H6	120.2	C22—C23—H23	120.0
C5—C6—H6	120.2	C23—C24—C25	123.8 (5)
C8—C7—C12	117.8 (4)	C23—C24—N1	117.5 (5)
C8—C7—Sn1	122.1 (3)	C25—C24—N1	118.6 (5)
C12—C7—Sn1	120.0 (3)	C20—C25—C24	115.1 (4)
C9—C8—C7	120.7 (5)	C20—C25—C26	120.7 (4)
C9—C8—H8	119.6	C24—C25—C26	124.2 (4)
C7—C8—H8	119.6	C25—C26—H26	109.5
C8—C9—C10	121.2 (6)	C25—C26—H26B	109.5
C8—C9—H9	119.4	H26—C26—H26B	109.5
C10—C9—H9	119.4	C25—C26—H26C	109.5
C11—C10—C9	119.3 (6)	H26—C26—H26C	109.5
C11—C10—H10	120.3	H26B—C26—H26C	109.5
C9—C10—H10	120.3	O5—C27—H27A	109.5
C10—C11—C12	120.6 (6)	O5—C27—H27B	109.5
C10—C11—H11	119.7	H27A—C27—H27B	109.5
C12—C11—H11	119.7	O5—C27—H27C	109.5
C7—C12—C11	120.3 (6)	H27A—C27—H27C	109.5
C7—C12—H12	119.8	H27B—C27—H27C	109.5
			
C7—Sn1—O1—C19	53.5 (3)	C1—Sn1—C13—C18	−35.2 (4)
C1—Sn1—O1—C19	−73.7 (3)	O1—Sn1—C13—C18	61.5 (3)
C13—Sn1—O1—C19	171.5 (3)	O5—Sn1—C13—C18	−118.4 (3)
C7—Sn1—O5—C27	46.3 (4)	C7—Sn1—C13—C14	−15.7 (4)
C1—Sn1—O5—C27	172.6 (4)	C1—Sn1—C13—C14	151.3 (3)
C13—Sn1—O5—C27	−72.1 (4)	O1—Sn1—C13—C14	−112.0 (3)
C7—Sn1—C1—C6	−48.3 (4)	O5—Sn1—C13—C14	68.2 (3)
C13—Sn1—C1—C6	145.8 (3)	C18—C13—C14—C15	−0.7 (7)
O1—Sn1—C1—C6	55.7 (4)	Sn1—C13—C14—C15	172.7 (4)
O5—Sn1—C1—C6	−128.6 (4)	C13—C14—C15—C16	0.1 (8)
C7—Sn1—C1—C2	132.7 (3)	C14—C15—C16—C17	1.6 (9)
C13—Sn1—C1—C2	−33.2 (4)	C15—C16—C17—C18	−2.6 (9)
O1—Sn1—C1—C2	−123.4 (3)	C14—C13—C18—C17	−0.3 (7)
O5—Sn1—C1—C2	52.4 (3)	Sn1—C13—C18—C17	−174.1 (4)
C6—C1—C2—C3	2.9 (7)	C16—C17—C18—C13	2.0 (8)
Sn1—C1—C2—C3	−178.1 (4)	Sn1—O1—C19—O2	3.2 (6)
C1—C2—C3—C4	−1.5 (9)	Sn1—O1—C19—C20	−176.5 (3)
C2—C3—C4—C5	−0.5 (10)	O2—C19—C20—C21	−64.5 (5)
C3—C4—C5—C6	1.2 (11)	O1—C19—C20—C21	115.2 (4)
C2—C1—C6—C5	−2.2 (7)	O2—C19—C20—C25	116.6 (5)
Sn1—C1—C6—C5	178.8 (4)	O1—C19—C20—C25	−63.7 (5)
C4—C5—C6—C1	0.2 (9)	C25—C20—C21—C22	−0.5 (6)
C1—Sn1—C7—C8	−23.2 (4)	C19—C20—C21—C22	−179.4 (4)
C13—Sn1—C7—C8	142.3 (4)	C20—C21—C22—C23	−0.7 (7)
O1—Sn1—C7—C8	−127.3 (4)	C21—C22—C23—C24	0.8 (7)
O5—Sn1—C7—C8	56.6 (4)	C22—C23—C24—C25	0.2 (7)
C1—Sn1—C7—C12	155.6 (3)	C22—C23—C24—N1	177.2 (5)
C13—Sn1—C7—C12	−38.8 (4)	O4—N1—C24—C23	48.4 (8)
O1—Sn1—C7—C12	51.5 (4)	O3—N1—C24—C23	−126.4 (6)
O5—Sn1—C7—C12	−124.5 (4)	O4—N1—C24—C25	−134.5 (6)
C12—C7—C8—C9	−0.1 (7)	O3—N1—C24—C25	50.7 (8)
Sn1—C7—C8—C9	178.8 (4)	C21—C20—C25—C24	1.4 (6)
C7—C8—C9—C10	0.3 (9)	C19—C20—C25—C24	−179.7 (4)
C8—C9—C10—C11	0.0 (10)	C21—C20—C25—C26	−179.5 (5)
C9—C10—C11—C12	−0.4 (10)	C19—C20—C25—C26	−0.6 (6)
C8—C7—C12—C11	−0.3 (7)	C23—C24—C25—C20	−1.3 (7)
Sn1—C7—C12—C11	−179.2 (4)	N1—C24—C25—C20	−178.3 (4)
C10—C11—C12—C7	0.6 (9)	C23—C24—C25—C26	179.6 (5)
C7—Sn1—C13—C18	157.8 (3)	N1—C24—C25—C26	2.6 (7)

### Hydrogen-bond geometry (Å, °)

**Table:**

*D*—H···*A*	*D*—H	H···*A*	*D*···*A*	*D*—H···*A*
O5—H5···O2^i^	0.85 (1)	1.87 (2)	2.676 (4)	158 (5)

Symmetry codes: (i) −*x*+3/2, *y*−1/2, −*z*+3/2.
